# CRITICAL VIEW OF SAFETY: A PROSPECTIVE SURGICAL AND PHOTOGRAPHIC ANALYSIS IN LAPAROSCOPIC CHOLECYSTECTOMY – DOES IT HELP TO PREVENT IATROGENIC LESIONS?

**DOI:** 10.1590/0102-6720202400034e1827

**Published:** 2024-10-25

**Authors:** Ana Carolina Buffara BLITZKOW, Alexandre Coutinho Teixeira de FREITAS, Júlio Cezar Uili COELHO, Antonio Carlos Ligocki CAMPOS, Marco Aurelio Raeder da COSTA, Victor Assad BUFFARA-JUNIOR, Jorge Eduardo Fouto MATIAS

**Affiliations:** 1 Universidade Federal do Paraná, Postgraduate Program in Surgical Clinic – Curitiba (PR), Brazil; 2 Universidade Federal do Paraná, Department of Surgery – Curitiba (PR), Brazil; 3 Hospital Santa Cruz – Rede D’or, Department of Surgery – Curitiba, Paraná (PR), Brazil; 4 Pilar Hospital, Department of Surgery – Curitiba (PR), Brazil

**Keywords:** Cholecystectomy laparoscopic, Cholelithiasis, Common bile duct, Cholecystectomy, Cholecystitis, acute, Colecistectomia laparoscópica, Colelitíase, Ducto colédoco, Colecistectomia, Colecistite aguda

## Abstract

**BACKGROUND:**

The incidence of biliary duct injuries remains higher in laparoscopic cholecystectomy (LC) in comparison to open surgery. The Critical View of Safety (CVS) was introduced by Strasberg as a strategy for reducing this catastrophic complication. AIM: The aim of this study was to evaluate how often an adequate CVS is achieved during LC, the determining factors for its success, and the associated surgical outcomes.

**METHODS:**

This is a prospective study. CVS photographs of all patients who underwent LC by the same surgeon between 2020 and 2023 were taken. Success in achieving CVS was analyzed by the surgeon herself and posteriorly by hepatobiliary specialists. Patients were classified into two groups: CVS achieved and CVS not achieved. Finally, multivariable logistic regression was used to examine the association between preoperatory factors and surgical complications.

**RESULTS:**

Three hundred and nine consecutive patients were submitted to LC. There were 73.5% elective CL and 26.5% acute cholecystitis. The age ranged from 14 to 87 years, and 76.8% were female. The median body mass index was 26.7. Previous abdominal surgeries were present in 64%, and 26% were obese. The CVS was achieved in 79.9% of the patients, and there were no surgical complications in this group. The factors associated with nonachievement were acute cholecystitis (p=0.007), male sex (p=0.014), and previous surgeries (p=0.021). Three patients needed a subtotal cholecystectomy due to severe inflammation. There was no statistical correlation between the identification of CVS and surgical complications.

**CONCLUSIONS:**

The CVS is achieved in most patients. Acute cholecystitis, male sex, and previous abdominal operations are associated with difficulties in obtaining CVS.

## RESUMO

**RACIONAL:**

A incidência das lesões de vias biliares (LVB) permanece maior na colecistectomia laparoscópica (CL) em comparação à colecistectomia aberta. A Visão Crítica de Segurança (VCS) foi introduzida por Strasberg como estratégia para redução dessa catastróficas complicação.

**OBJETIVOS:**

Avaliar com que frequência a VCS adequada é alcançada durante a CL, os fatores determinantes e as complicações cirúrgicas associadas.

**MÉTODOS:**

É um estudo prospectivo. Fotografias da VCS de todas as CL realizadas consecutivamente pelo mesmo cirurgião entre 2020 e 2023 foram feitas. O sucesso na obtenção da VCS foi analisado pela própria cirurgiã e, posteriormente, por cirurgiões hepatobiliares. Os pacientes foram classificados em dois grupos: VCS obtida e VCS não obtida. Foi utilizada regressão logística multivariável para avaliar a associação entre fatores pré-operatórios e complicações cirúrgicas em ambos os grupos.

**RESULTADOS:**

Trezentos e nove pacientes foram submetidos a CL. Desses, 73,5% foram cirurgias eletivas e 26,5% apresentavam colecistite aguda. A idade variou entre 14 a 87 e 76,8% eram mulheres. O IMC médio foi 26,7. Cirurgias abdominais prévias ocorreram em 64%. A VCS foi obtida em 79,9% dos pacientes e não houve complicações cirúrgicas neste grupo. Os fatores associados à não obtenção da VCS foram colecistite aguda (p=0,007), sexo masculino (p=0,014) e cirurgias abdominais prévias (p=0,021). Três pacientes necessitaram de colecistectomia subtotal devido à inflamação grave. Não houve correlação estatística entre a obtenção da VCS e complicações cirúrgicas.

**CONCLUSÕES:**

A VCS é obtida na maioria dos pacientes. Colecistite aguda, sexo masculino, e cirurgias abdominais prévias estão associados a dificuldades na obtenção da VCS.

## INTRODUCTION

Cholelithiasis is a very common disease, with a prevalence of about 10% in the general population of Brazil and most Western countries, and reaches almost 28% in persons older than 70 years^6^. Laparoscopic cholecystectomy (LC) is one of the most common procedures by general surgeons worldwide^18^.

The first LC was performed in 1985 by Prof Dr. Erich Mühe of Böblingen, Germany^24^. A few years later, LC became the gold standard treatment for symptomatic gallstones due to several advantages. Compared with open cholecystectomy, this procedure has several benefits, including less postoperative pain and infections, reduced hospital stay, and a shorter recovery period, resulting in lower costs and better cosmetic results^3^^-^ 11 ^-^ 27 ^-^ 32. Most LCs are performed in ambulatory settings in several countries, including Brazil^7^.

Unfortunately, since the widespread use of the laparoscopic technique, the incidence of BDI remains 0.3–0.8%, compared to 0.2% in open operations^17^^-^ 23 ^-^ 35. In response to the increase in biliary injury associated with LC, Strasberg introduced 1995 the term “Critical View of Safety” (CVS). Many surgeons recognize the CVS as the best practice, consisting of a secure identification method of the cystic duct and cystic artery during LC^35^. However, the incidence of biliary injuries has not significantly decreased in the past decade^25^. One possible reason may be that although CVS is recommended, it is not used routinely by many surgeons^12^^-^ 20.

This study aims to describe how often an adequate CVS is achieved during LC in a series of consecutive LCs performed by the same surgeon. In addition, potential confounding factors that might impact the ability to obtain an adequate CVS and the incidence of surgical complications are also analyzed.

## METHODS

This is a prospective study. All the patients who underwent consecutive LC, by the same surgeon, from August 2020 to February 2023, were included. The surgeries included elective and emergency cases in two hospitals in Curitiba, Paraná, Brazil. This study was approved by the Ethical Committee of the University Hospital, Universidade Federal do Paraná (number 61705522.5.0000.0096).

Indications for elective LC included: symptomatic cholecystolithiasis, symptomatic cholesterolosis, or biliary polyps^5^^-^ 13 ^-^ 14. The indication for emergency LC was acute cholecystitis. In all cases, the diagnosis of gallbladder disease was confirmed by either ultrasonography, computerized tomography, or magnetic resonance imaging.

Acute cholecystitis was defined according to the Tokyo guidelines criteria^44^. All cases of acute cholecystitis were operated on as soon as possible, within 24 h. LCs of patients following emergency admissions who did not have criteria for acute cholecystitis were classified as elective LC (e.g., biliary pancreatitis, choledocholithiasis, and cholangitis). In these cases, the LCs were usually done in the same admission but as an elective procedure.

**Figure 1:**
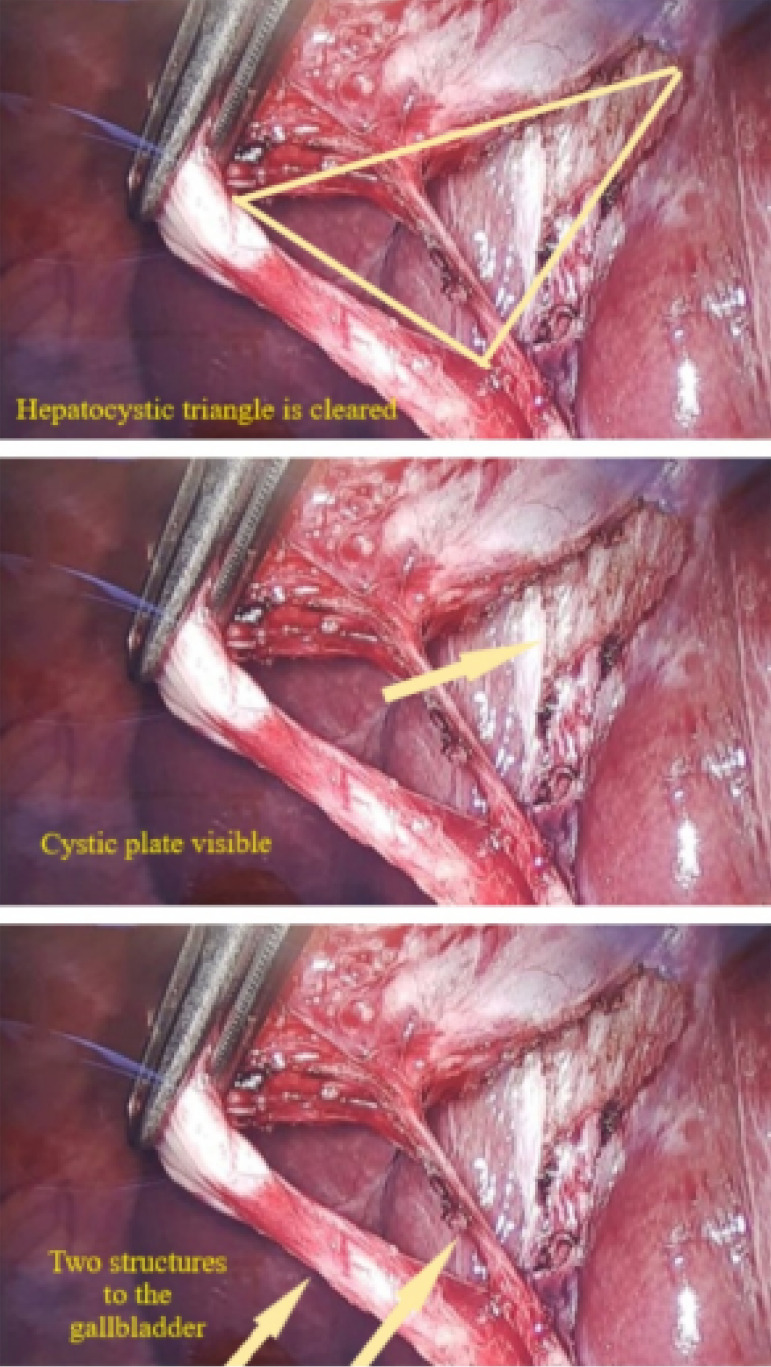
Adequate critical view of safety.

LC was performed using a four-port standard technique. In all cases, the surgeon tried to find CVS and registered with a single or double photograph of the hepatocystic triangle. Photos of the operation and patient data were recorded. Figure 1 shows the three requirements for CVS. 

Photographs of all surgeries were stored in a camera. The images were randomly divided among four professors of surgery with a large experience in hepatobiliary surgery in order to determine if adequate CVS was obtained by the surgeon who performed the LC. The images were rated using a 6-point CVS assessment tool (Table 1)^28^. Five or more points were considered a satisfactory CVS. The evaluators were blinded to patient data.

**Table 1:** Criteria for assessing photographs of the critical view of safety^28^.

Two structures connected to the gallbladder	
2 points	Two structures can immediately and clearly be seen connecting to the gallbladder.
1 point	Two structures can be seen connecting to the gallbladder, but there is some overlap of duct and artery or a technical feature, such as poor lighting or lack of color contrast, that interferes with clarity of determination. Therefore, the photograph requires study to make assessment.
0 points	Due to overlap or technical issues, two separated cystic structures cannot be seen.
**Cystic plate**	
2 points	Cystic plate is immediately clearly visible to approximately its bottom one-third.
1 point	Cystic plate is visible but overlapped by other structures so that it is not optimally seen or an insufficient amount of the plate is shown. The photograph requires study to make assessment.
0 points	Cystic plate not visible due to positioning, light, obstruction of view by instruments, or coverage with clot.
**Clearance of hepatocystic triangle**	
2 points	Hepatocystic triangle is cleared of tissue so that the visibility of cystic structures and plate is completely unimpeded, but also so that the viewer can be immediately certain that no other structures are in the triangle.
1 point	Somewhat less than the whole triangle can be clearly seen or technical issues reduce the ability to see optimally. The photograph requires study to make assessment.
0 points	Tissue in the triangle obscures the view of cystic structures and plate and does not allow the conclusion that there are no other structures in the triangle, or technical issues prevent the determination of how well cleared the triangle is.

The patients were divided into two groups: one group with satisfactory achievement of CVS and the second with no achievement of CVS. The following clinical data were compared among the groups: age, gender, body mass index (BMI) (patients with a BMI over 30 were classified as obese), presence of diabetes (yes or no), previous abdominal surgeries (yes or no), information if acute or elective cholecystitis, and postoperative complications.

Quantitative variables were described using mean and standard deviation, while qualitative variables were described using absolute and relative frequencies. Comparisons between groups were performed using Pearson’s χ^2^ and Student’s t-test. To identify factors associated with CVS, logistic regression was performed with sex, age, type of surgery (chronic or acute), diabetes mellitus, BMI, and previous operations as covariates and surgical complications. The odds ratio (OR) and their respective 95% confidence intervals (95%CI) were reported. All statistical analyses were done using the R software, and a p-value below 0.05 was considered statistically significant.

## RESULTS

A total of 309 patients were submitted to LC. According to the surgeon’s perception, the CVS was adequately achieved in 306 of 309 cases (99%). In three patients (0.97%) for whom the CVS was not obtained, the surgeon performed subtotal cholecystectomy (STC) to avoid BDI. The professor’s evaluations and scores about the achievement or not of the CVS by the surgeon are shown in Table 2. They were different from the surgeon’s impressions described in the surgical notes in 59 patients (19%). The professor considered the CVS satisfactory (five points or more) in 247 patients (79.9%).

**Table 2:** Evaluating professor’s scores given to the critical view of safety.

Critical view of safety			
Evaluating professor	Sample n=309	Yes n=247* (79.9%)	No n=62* (21.1%)
Professor 1	30	28 (93.33)	2 (6.66)
Professor 2	219	171 (78.08)	48 (21.91)
Professor 3	30	25 (83.33)	5 (16.66)
Professor 4	30	23 (76.66)	7 (23.33)

Mean: standard deviation.

* n (%).

Table 3 shows the demographic and clinical characteristics of the patients. The most common indications for elective LC were chronic cholecystitis with cholelithiasis, biliary polyps, or cholesterolosis (73% of the cases). Acute cholecystitis occurred in 26.5% of the cases. Among patients who underwent cholecystectomy, 76.6% were female. The age ranged from 14 to 87 years, with a median age of 43 years. The median BMI was 26.7, and 9.4% of the patients had diabetes. In addition, 64% of the patients had previous abdominal operations, and 26% were obese (BMI>30). Table 3 summarizes the univariate comparison of the clinical characteristics and outcomes between patients for whom the CVS was achieved (n=247, 79.9%) and those who did not achieve CVS (n=62, 20.1%). Surgical complications occurred only in the group in which the CVS was not achieved (Table 3).

**Table 3:** Sample characteristics and critical view of safety.

Characteristics		Critical view of safety			p-value†
		Sample n=309* (%)	No n=62* (%)	Yes n=247* (%)	
Sex (n=309)					
	Female	237 (76.7)	42 (67.7)	195 (78.9)	0.062
	Male	72 (23.3)	20 (32.3)	52 (21.1)	
	Age	43.0 (13.5)	43.3 (13.3)	43.0 (13.6)	0.874
Type of LC					
	Emergency	82 (26.5)	26 (41.9)	56 (22.7)	0.002
	Elective	227 (73.5)	36 (58.1)	191 (77.3)	
	Diabetes mellitus	29 (9.4)	8 (12.9)	21 (8.5)	0.288
	BMI	26.7 (5.0)	27.8 (5.2)	26.4 (4.9)	0.074
	Obesity	69 (22.3)	16 (25.8)	53 (21.5)	0.462
	Previous operation	200 (64.7)	43 (69.4)	157 (63.6)	0.393
	Surgical complications	3 (1.0)	3 (4.8)	0 (0.0)	0.008
	Subtotal cholecystectomy	3 (1.0)	3 (4.8)	0 (0.0)	0.008
	Biliary leak	2 (0.6)	2 (3.2)	0 (0.0)	0.040

Mean: standard deviation; LC: laparoscopic cholecystectomy.

* n (%);

† Pearson’s χ^2^ test;

Welch two-sample t-test; Fisher’s exact test.

The multivariate logistic regression analyses revealed that elective cholecystectomy was associated with increased odds of achieving a CVS (OR=2.37; 95%CI 1.26–4.42; p=0.007, p<0.05) (Table 4). The incidence of not obtaining the CVS was significantly higher for acute cholecystitis patients (32%) than for elective patients (16%) (p=0.002, p<0.05) (Table 3).

**Table 4:** Logistic regression for critical view of safety.

Characteristic		OR	95%CI	p-value
Sex				
	Female	-	-	
	Male	0.40	0.19–0.83	0.014
Age		1.01	0.98–1.04	0.426
Type				
	Acute	-	-	
	Chronic	2.37	1.26–4.42	0.007
Diabetes mellitus		0.83	0.31–2.44	0.722
BMI		0.95	0.89–1.01	0.080
Any previous abdominal operations		0.41	0.18–0.86	0.021
Surgical complications				
	No	-	-	
	Yes	0.00		0.982

OR: odds ratio; CI: confidence interval; BMI: body mass index.

Decreased odds of achieving a CVS were observed in males (OR=0.48; 95%CI 0.23–0.99; p=0.045, p<0.05) and in patients with previous abdominal operations (OR=0.41; 95%CI, 0.18–0.86; p=0.021, p<0.05). Among the other risk factors analyzed (Table 4), no significant statistical correlation with nonachievement of CVS was found between obesity (p=0.08, p>0.05), diabetes (p=0.722, p>0.05), and age (p=0.426, p>0.05). In the multivariate logistic regression analyses, there was no statistical correlation between the identification of CVS and surgical outcomes or complications (p=0.982, p>0.05).

The incidence of surgical complications is shown in Tables 3 and 4. In three patients, according to the surgeon’s perception, the obtention of the CVS was not possible due to severe fibrosis and inflammation of the hepatocystic triangle. Hence, a subtotal fenestrating cholecystectomy^36^ was done in these three cases. All the stones were removed during this procedure, and the abdominal cavity was drained with a laminar drain. Two (66.6%) of the three patients submitted to STC developed biliary leak. Hence, biliary fistula occurred in 0.64% of the 309 LC. Of the patients with a biliary fistula, one had self-limiting drainage, and no additional interventions were indicated. For the other patient with the biliary fistula, an endoscopic retrograde cholangiopancreatography (ERCP) with papillotomy was performed, and the fistula closed three days after the procedure. The third patient submitted to STC had an uneventful postoperative recovery with no biliary leak. None of the three patients presented with recurrent symptoms so far. Therefore, there were no differences in the complication rates when comparing the group in which CVS was achieved to those in which CVS was not achieved (p=0.08, p>0.05).

During ambulatory follow-up, one patient was diagnosed with pulmonary embolism on the sixth postoperative day. He was treated with rivaroxaban and had a good clinical evolution. No major BDI, conversion to open surgery, or death was observed in this study.

## DISCUSSION

The BDI rate associated with LC is still higher than that of open cholecystectomy^23^^-^ 25. LC is the most frequent cause of BDI, with an estimated 3000 BDI occurring annually in the United States due to cholecystectomies^9^. Although BDI is relatively rare, the impact is significant, given the high volume of LCs. Biliary injuries are associated with high morbidity and higher costs. They are a frequent cause of legal claims against surgeons. The results of iatrogenic BDI may be severe, leading to liver transplant or even death, depending on the type of lesion and time of recognition^26^.

According to the literature, there are multiple factors contributing to BDI: anatomical variants of the biliary tract, obesity, previous surgery, inexperience of the surgeon, an underlying liver disease, and acute cholecystitis^21^. The severity of gallbladder disease is the most important prognostic factor of LC. BDI occurs three times more often in patients with severe local conditions due to active acute cholecystitis than without inflammation^38^. Not surprisingly, our study observed that the incidence of not obtaining the CVS was significantly higher for acute cholecystitis patients (p=0.002, p<0.05). CVS was obtained in 84% of elective patients and 68% of patients in the emergency setting.

The role of gender as a risk factor for LC is controversial in the literature^8^. In previous studies, the preoperative criteria for failing to achieve the CVS included male sex, age over 60 years, obesity, and previous abdominal surgeries^19^^-^ 28 ^-^ 37. Our study also found that male sex was associated with decreased odds of achieving a CVS. We also found that previous abdominal operations were related to difficulties in obtaining CVS. Contrary to other authors, we did not find the difference between groups regarding age and the presence of obesity, defined as BMI>30 kg/m^2^ 19 ^-^ 28 ^-^ 31. These different findings may be due to the small number of LCs evaluated in the present study.

Most major BDIs tend to occur by misidentification of anatomical structures. One study stated that misinterpretation of anatomy was the primary factor cited by 92.7% of surgeons^25^^-^ 39. Therefore, reliable surgical techniques for LC are essential for surgeons. The CVS is a great model to avoid misidentification and to reduce the incidence of BDI incidence^42^. Many biliary surgeons use CVS as the method of identification used to train surgical residents in many institutions. Many surgeons still prefer the infundibular technique, which consists of dissecting the gallbladder from its neck upward after dissecting the cystic duct and the cystic artery. A study found that 56% of surgeons prefer the infundibular approach, 27% used the CVS method, and 27% used the intraoperative image^9^. The infundibular method requires less dissection. However, it is recommended that it should not be used as the exclusive method of cystic duct identification because it may be unreliable. The CVS technique is considered superior to the infundibular technique^9^^-^ 34.

Several studies suggest that a large proportion of surgeons are unable to describe the definition of CVS. Although CVS is very publicized, it is still underutilized^9^^-^ 12. The first CVS criterion is that the hepatocystic triangle is cleared of all fibrous and fat tissues. Second, the lower part of the gallbladder is dissected off the liver to expose one-third of the cystic plate. The third criterion of the CVS is that two and only two structures enter the gallbladder. All three criteria must be performed to obtain the CVS (Figure 1). The Calot triangle and hepatocystic triangle (or modern Calot) are different: The cranial border in Calot’s triangle is determined by the cystic artery, and the cranial border in the hepatocystic triangle is defined by the lower margin of the liver, which provides a better and more constant boundary safety due to the wide range of anatomic variations of the cystic artery^1^. CVS does not require that either the common bile duct or the common hepatic duct be visualized^33^.

Several authors have demonstrated that the correct establishment of CVS effectively reduces biliary lesions. The literature suggests that the obtention of the CVS could reduce the BDI rate from 0.4% to nearly 0%^2^^-^ 4 ^-^ 16 ^-^ 19 ^-^ 29 ^-^ 43. In addition, studies also found that the operative time is significantly reduced with the CVS technique^45^. Our study found no complications or biliary injuries in 247 cholecystectomies that correctly assessed the CVS.

The CVS is not always achievable, for example, in the presence of severe inflammation or anatomic variations. The impossibility of obtaining CVS was associated with a higher operative difficulty grade and morbidity^19^. During a difficult gallbladder in which the CVS cannot be attained, various strategies should be used: intraoperative biliary imaging like cholangiogram, fluorescence, or laparoscopic ultrasound; dissection consistently above Rouvière’s sulcus; conversion to open cholecystectomy; fundus-first technique or getting help from a colleague. In addition, a bailout procedure can be considered if the hepatocystic triangle cannot be safely dissected, for example, a cholecystostomy to reduce the acute condition, an STC, or even abort the LC and refer the patient to a tertiary center^15^^-^ 31 ^-^ 41 ^-^ 42 ^-^ 44.

STC is the most frequent bailout alternative employed in difficult LC (1.9%)^15^. It is an essential strategy for surgeons in complex intraoperative situations at high risk of postoperative complications^15^. During an STC, the removal of all gallbladder stones should be performed. In the reconstituting approach, the neck of the gallbladder is sutured or stapled. Contrarily, in the fenestration procedure, the gallbladder neck is left open (the cystic duct can be sutured from within the lumen of the gallbladder or left open)^10^^-^ 36. In our study, three patients were submitted to fenestrating STC due to the impossibility of achievement of CVS. While it remains unclear which subtype is the best option for the difficult gallbladder^10^, some authors recommend fenestrating STC as the most definitive bailout procedure. Although this procedure seems to be related to a higher rate of postoperative bile leak, cases where the gallbladder is “reconstituted” are associated with more recurrence of biliary events^30^^-^ 40.

Some studies have demonstrated that operative notes are less reliable than photographs or videos^22^. Image recording of the CVS by videos or photos is recommended as it allows studies to determine whether CVS is being attained and provides insight into why these injuries occur. Sanford et al. described the doublet photo method, which consists of photos of the anterior and posterior views of the CVS. This could be used to reliably confirm that the CVS has been reached, using a six-point scale to assess the achievement of the CVS (Table 1)^28^. We used this method — described by Sanford. It was easy, fast, cheap, and accessible. The operation room (O.R.) nurse took the photos of CVS with the surgeon’s camera.

In the surgeon’s perception, the CVS was achieved in 99% of the LC. The professor’s evaluation reported 79.9%. We attributed this difference to two leading causes. First, the perception of the surgeon may be wrong in the self-assessment, as already cited in previous studies^9^^-^ 20. Nissjen studied 65 videos from complications of LC, and only 11% of videos confirmed the CVS correctly, although, in surgical reports, 80% said the CVS was achieved^20^. Second, the evaluating professors said they had difficulty evaluating a minority of photos. Photography has limitations^28^: some had low quality and poor focus, were cloudy, and contained blood, bile, or pus; angles from where these specific images were taken made it difficult to identify the structures. These factors may have contributed to low scores in some patients. Some groups have suggested that video images are superior to photos for accurately judging CVS by independent surgeons after the surgery, although they are more difficult to store than photos^22^. We may consider using videos in our next cholecystectomies and further studies.

## CONCLUSIONS

The CVS is adequately obtained in most patients subjected to LC. The factors associated with difficulty achieving CVS are acute cholecystitis, male sex, and previous abdominal surgery.
